# Etiologies and Outcomes of Acute Liver Failure in a Spanish Community

**DOI:** 10.1155/2013/928960

**Published:** 2013-08-19

**Authors:** Emilio Fábrega, Miguel Ángel Mieses, Alvaro Terán, Irene Moraleja, Fernando Casafont, Javier Crespo, Fernando Pons-Romero

**Affiliations:** Gastroenterology and Hepatology Unit, University Hospital “Marqués de Valdecilla”, Instituto de Formación e Investigación Marqués de Valdecilla (IFIMAV), Avenida Valdecilla s/n., Santander, 39008 Cantabria, Spain

## Abstract

Previous retrospective study (1992 to 2000) performed in Spain showed that drug toxicity, viral hepatitis, and indeterminate etiology were the most prevalent causes of acute liver failure (ALF). In the last decade, there is no information about ALF in our country. For these reasons we analyze retrospectively, in a ten-year period (2000 to 2010), the presumed causes, clinical characteristics, course, and outcome of ALF in a Spanish community. Causes of ALF were indeterminate in 4 patients (24%), acute hepatitis B infection in 4 patients (24%), drug or toxic reactions in 4 patients (24%), including one case of acetaminophen overdose, followed by miscellaneous causes. The overall short-term survival (6 weeks after admission) was 65%. Liver transplantation was performed in 11 patients with a survival of 82%. Despite fulfilling criteria, 2 patients were not transplanted because of contraindications; they both died. In summary, acute hepatitis B and indeterminate cause are still being the most frequent causes of ALF in our region, and patients with ALF have an excellent chance of survival after emergency liver transplantation. Acetaminophen overdose still represents a very rare cause of ALF in our community.

## 1. Introduction

Acute liver failure (ALF) is a clinical syndrome, in which there is an acute insult in a patient without a known preexisting liver disease that leads to a rapid loss of liver function, characterized mainly by hepatic encephalopathy (HE), jaundice and coagulopathy [1]. The prognosis of these patients was very poor until the introduction of liver transplantation (LT) for the treatment of this disease in the last decades, improving their survival significantly [1, 2]. Current results of LT are very good considering the natural history of the disease, the multiorgan involvement, the emergency context, and the lack of other effective therapies [3].

An important aspect of ALF that needs further clarification is the varying composition of ALF causes among different geographic regions [2, 4]. One retrospective study from Spain summarized 267 ALF cases observed from 1992 to 2000 [5]. Our center participated in this study and drug toxicity, viral hepatitis and indeterminate etiology were the most prevalent causes of ALF [5]. In the last decade, there are no published studies about ALF in our country. For that reason, in our hospital, we decided to determine retrospectively if in the last ten years there have been changes in the etiology, clinical course, and outcome in patients with ALF.

## 2. Patients and Methods

### 2.1. Enrollment of Patients and Definitions of Severe Acute Liver Injury and Acute Liver Failure

A retrospective, longitudinal study was carried out at Marqués de Valdecilla University Hospital (Santander, Spain). The hospital medical records of patients who underwent the diagnosis of acute hepatitis between January 2000 and December 2010 were reviewed and validated manually. Severe acute liver injury was defined as acute (<12 weeks) liver dysfunction leading to an international normalized ratio >1.5, but without documented level of HE. Overt ALF was defined as evidence of coagulation abnormality, usually an international normalized ratio ≥1.5, and any degree of mental alteration (HE) in a patient without preexisting cirrhosis and with an illness of <26 weeks of duration [3]. 

ALF was classified as fulminant when HE appeared within the first two weeks after the onset of jaundice and as subfulminant when appearing between weeks 3 and 8 [2]. In addition, patients were also classified according to O'Grady classification as having hyperacute, acute, or subacute impairment [6]. 

### 2.2. Patient Collective and Parameter Evaluation

The medical records of these patients were searched after institutional review board approval was obtained for the following data fields: demographic features, date of diagnosis, suspected cause of ALF, exposed to an NSAIDs or OTC, extra hepatic complications during the admission; such as hypoglycemia, infections, gastrointestinal bleeding, renal failure, and cerebral edema; grade of HE, date of transplantation, time in waiting list, and contraindication for transplantation. Short-term outcomes including survival with or without LT (spontaneous survival) and death were determined at 6 weeks after study admission. Long-term survival (one-year survival) in transplanted patients was reviewed.

In all patients, ALF etiologies were based on accepted diagnostic criteria including clinical history, laboratory values, imaging studies, and subsequent pathological examination of liver explants consistent with massive or submassive necrosis. ALF was considered to be indeterminate when clinical laboratory evaluations (including toxicological screening, serological markers for viral hepatitis A, B, C, D, E, autoantibodies, and metabolic as Wilson disease) and imaging studies were inconclusive. Hepatic encephalopathy was graded on a standard scale of 1–4 as described previously [7]. King's College criteria and Clichy's criteria were determined as described [2, 8, 9]. Cerebral edema was diagnosed by clinical and imaging data. No patient was monitored for intracranial pressure. All patients were treated similarly with standard supportive treatment and strict monitoring in the ward, or if required in the intensive care unit.

### 2.3. Statistical Analysis

The results were analyzed with the SPSS 15.0 computer software package (Statistical Package for Social Sciences, Inc., Chicago. IL) Continuous variables were summarized as means or as medians and ranges. Categorical variables were compared with the chi-square test and Fisher's exact test.

## 3. Results

Our hospital has been a tertiary academic center with experience in LT since 1990, and we are the hospital of reference for LT for two autonomous community (Cantabria and La Rioja), covering a population of one million inhabitants.

### 3.1. Study Population

**Figure 1:**
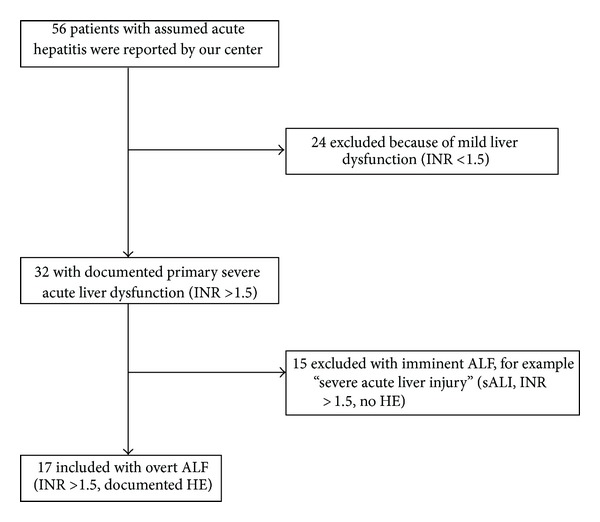
Enrollment of study patients.

During the study period (January 1, 2000 to December 31, 2010), 56 patients were admitted in our institution with the diagnosis of “acute hepatitis” with or without coagulopathy and/or HE. From these patients, 24 patients had a mild acute hepatitis, 15 patients severe acute liver injury, and only 17 patients met criteria for ALF (Figure 1). We are aware that the retrospective design of our study could be a pitfall when calculating the incidence of the disease.

### 3.2. Demographic Characteristics and Clinical Data

All patients were Spanish.Of the 17 patients with ALF, 11 (65%) were women. The median age of the group was 45 years (range 17 to 87 years) with 35% of patients younger than 40 years at presentation; women were younger than men (with a mean age of 40 versus 53 years, resp.).

At presentation, the absence of HE was the most frequent finding (58%), with only one patient (6%) presenting deep coma (grade IV HE), although this relation reversed during followup (35% of patients developed grade IV, whereas no patients remain without HE). The mean interval from onset of symptoms to HE was 18 days (range 1–84 days).

According to Bernau classification, 76% (13 patients) had a fulminant, and 24% (4 patients) had a subfulminant liver failure. On the other hand, according to, O' Grady, 53% (9 patients) were hyperacute, 24% were acute (4 patients), and 24% (4 patients) were subacute liver failure (Table 1).

### 3.3. Causes of ALF

**Figure 2:**
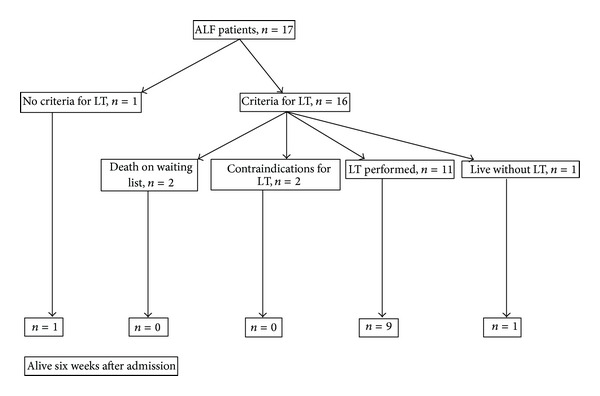
Outcome of 17 patients with acute liver failure.

Figure 2 and Table 1 display the presumed causes of ALF and the outcome data for all these patients. No patients were exposed to an NSAID or OTC within 30 days before the onset of clinical symptoms. Acute hepatitis B and indeterminate causes were the most frequent causes of ALF, accounting for eight patients. Among these eight, four of them (24%) had acute hepatitis B, and the other four (24%) had indeterminate causes.

Drug or toxic reactions were responsible for four cases (24%) of ALF: one case of ecstasy/N-methyl-3,4-methylenedioxyamphetamine (MDMA), one case of antituberculosis drug induced hepatotoxicity, one case of antiandrogen therapy for prostate cancer, and one case of acetaminophen overdose with suicidal intent.

Finally, there was a miscellaneous group of five patients (29%), including two cases of autoimmune hepatitis presenting as ALF, one case of acute fatty liver pregnancy-associated, one case of mushroom poisoning, and one case of subfulminant hepatitis after biliopancreatic diversion for morbid obesity.

### 3.4. Extrahepatic Complications

Five patients (29%) presented medical complications at admission. However, during followup, the incidence of these complications increased to twelve patients (71%). At admission, the most frequent complication was renal failure. During followup, the most frequent complications were renal failure (29%) followed by hypoglycemia (23%), bacterial infection (12%), signs of cerebral edema (12%), gastrointestinal bleeding (12%), respiratory failure (6%), and multiorgan failure (6%).

### 3.5. Outcome

The overall survival was 65% (11 of 17 patients) at the end of the hospitalization (Figure 2). One patient was considered to not fulfill criteria for emergency liver transplantation (ELT) and survived without LT (Figure 2). From those who fulfill criteria for LT, 69% (11 of 16 patients) were transplanted; in 12% (2 patients), transplantation was contraindicated (one patient had prostate cancer, and the other was an elderly patient, both patients died), 6% (1 patient) ALF resolved spontaneously, and 12% (2 patients) died while waiting in transplantation list for cerebral edema and multiorgan failure (Figure 2).

The overall survival in patients who were transplanted was 82% (9 of 11 patients) at the end of the hospitalization. Two patients died after transplantation, one because of abdominal sepsis and the other because of biliary complications. Long-term survival rate was 82% in patients who were transplanted.

The median time from waiting list to transplantation was 35 hours (range 7 to 120 hours), and median time to death after admission was 22 days (range 2 to 35 days).

## 4. Discussion

ALF is the most common term applied to an unusual clinical syndrome resulting from rapid loss in hepatocyte function [1]. It occurs infrequently, affecting 2000 patients annually in the United States, and comprises approximately seven percent of liver transplants annually. The annual incidence does not appear to be increasing or decreasing at this time [10], and these data are similar in Europe countries [11, 12]. The current study analyzes the cause and outcome of ALF in the Spanish community. It should be stressed that the retrospective design of the study did not allow us to reach conclusive results.

ALF is a rare critical disease that occurs mainly in young adults, approximately in fourth decade of life [10–12]. In our study, we found that the mean age of presentation (45 years) and female/male ratio (1.8/1) were similar to previous studies [4, 5, 13–16]. In the present, it is uncertain why women are more sensitive to ALF.

The etiology of this condition varies among the geographical area. In the United States [4, 17], Europe, and United Kingdom [18] acetaminophen is the main cause of ALF in contrast to India and other Asian countries, in which viral hepatitis is the main cause [13, 14, 19, 20]. In our study, we found an extraordinarily low incidence of acetaminophen-related ALF, accounting for <6% of cases, which is similar to the previous published data of the Spanish population [5]. This figure (recently reviewed by Polson and Lee) [21] strongly contrasts with the recent experience in most Western countries, such as the United Kingdom [18], Ireland, the United States [4], Sweden, and Denmark. This might be due to underestimation of acetaminophen as a cause of ALF because of possible inaccurate retrospective data acquisition and lack of proper history-taking concerning acetaminophen ingestion [22]. It has been shown that half of acetaminophen overdoses are apparently unintentional [23] and that acetaminophen toxicity is in fact causing many cases of ALF not attributed to the drug without specific testing [24]. In addition, the French experience indicated that acetaminophen overdose causing ALF has been progressively increasing in the last years, but this has not occur in Spain, at least in our experience [25].

Two causes of ALF account for approximately half of the patients in our study: the first is so-called indeterminate, that accounts for approximately one third of ALF, as shown in Table 1, which is almost similar to the Spanish and Argentinean experience [5, 26]. Recently, the US ALF Study Group has described that acetaminophen adducts were detected in serum in some cases of indeterminate causes; thus, these ALF cases could be classified due to this compound [24]. Therefore, it could be argued that some of our cases were in fact due to acetaminophen, although ingestion of this drug was not detected. In the US study, patients in whom acetaminophen adducts were detected showed hyperacute courses, with high serum amine transferase (ALT) levels similar to classical acetaminophen overdose. Our experience indicated that the majority of cases considered indeterminate with hyperacute courses makes the ingestion of acetaminophen possible.

The second main cause of ALF in our series was fulminate hepatitis B virus infection, a figure that is similar to the previous Spanish experience [5]. The universal vaccination against this agent that started more than two decades ago in Spain should decrease hepatitis B virus infections. However, in our community, this program was introduced later in 1996. We think that this late introduction of the immunization program could explain our findings. Nonetheless, since 2006 we have not had any new cases of acute hepatitis B liver failure, leading us to think that the universal vaccination program is accomplishing its objective, and maybe in the following years our incidence of acute hepatitis B liver failure can be reduced and be similar to that of developed countries.

Most patients in our study showed low grades of HE; half of the cases arrived at our hospital with grade 0. This observation was probably caused by the campaign in our community that promotes early referral to our active liver transplant program in our hospital.

Emergency liver transplantation (ELT) has been performed in 65% of our patients with ALF, which is the highest percentage among Western countries [27], reflecting the best graft availability and the small number of acetaminophen ingestion cases, since this etiology has the best prognosis making transplant unnecessary [27]. The rapid access to ELT in Spain also accounts for the lowest risk of death while on the waiting list. In the US ALF Study, 30% of patients listed dead awaiting a graft; whereas, in Spain only 4% of the overall series and 7% of those listed dead [5, 27], and 51.5% of the ALF patients were transplanted within 24 hours [5].

The survival of patients with ALF has changed dramatically since the introduction of ELT. Before transplantation era, mortality was very high, with survival rates without ELT ranging from 13%–18% [16, 28, 29]. Nowadays, overall survival is between 50%–70%, short-term survival after ELT 70%–80% and one-year rates survival is 79% according to some series [4, 5, 10, 11, 15, 27]. In our study, overall survival, short-term, and one-year rates survival after ELT were similar.

In summary, we find that acute hepatitis B and indeterminate cause are still being the most frequent causes of ALF in our community and that patients with ALF have good chance of survival with transplantation.

## Figures and Tables

**Table 1:** Clinical characteristics of patients according to O'Grady classification.

	Hyperacute LF	Acute LF	Subacute LF
Patients, *n*	9	4	4
Mean age (range), y	49 (19–69)	41 (21–61)	38 (17–57)
Women, *n* (%)	6 (67%)	2 (50%)	3 (75%)
Cause of ALF, *n*			
Hepatitis B	2	2	0
Indeterminate	2	1	1
Drugs	2^a^	0	1^b^
Mushrooms	1	0	0
Acetaminophen	1	0	0
Pregnancy	1	0	0
Autoimmune	0	1	1
Other	0	0	1
Survived without transplant, *n* (%)	1 (11)	0	0
Survived with or without transplant, *n* (%)	4 (44%)	3 (75%)	3 (75%)
Received transplant, *n* (%)	4 (44%)	3 (75%)	4 (100%)

One case of antituberculosis drug, and one case of antiandrogen therapy^a^; ecstasy^b^.
